# Trade-off between allocation to reproductive ramets and rhizome buds in *Carex brevicuspis* populations along a small-scale elevational gradient

**DOI:** 10.1038/srep12688

**Published:** 2015-07-31

**Authors:** Xin-sheng Chen, Ya-fang Li, Yong-hong Xie, Zheng-miao Deng, Xu Li, Feng Li, Zhi-yong Hou

**Affiliations:** 1 Key Laboratory of Agro-ecological Processes in Subtropical Region, The Chinese Academy of Sciences, Hunan 410125, China; 2 Dongting Lake Station for Wetland Ecosystem Research, Institute of Subtropical Agriculture, The Chinese Academy of Sciences, Changsha 410125, China; 3 College of Life Science and Technology, Central South Forestry University, Changsha 410004, China

## Abstract

The trade-off between allocation to sexual and clonal reproduction in clonal plants is influenced by a variety of environmental factors; however, it has rarely been examined under field conditions. In this study, we investigated the trade-off between two modes of reproduction in *Carex brevicuspis* C. B. Clarke across a small-scale elevational gradient (21–27 m a.s.l.) at the Dongting Lake wetlands, China. The proportion of biomass allocated to and the density of reproductive ramets were higher at low than at intermediate and high elevations. In contrast, the proportion of biomass allocated to and the density of rhizome buds were lower at low than at intermediate and high elevations. Redundancy analysis showed that sexual reproduction was positively correlated with soil moisture content, soil organic matter, total phosphorus, and pH, and negatively correlated with elevation and ramet density. Our findings suggested that allocation to sexual reproduction is favored in disturbed habitats with fertile soils, whereas allocation to vegetative propagation is favored in stable and competitive habitats. Trade-off between allocation to sexual reproduction and vegetative propagation along an elevational gradient might be a reproductive strategy of *C. brevicuspis* to adapt to the water level fluctuations in wetland habitats.

Clonal plants are widespread across all biomes and biogeographical regions, particularly in cold, wet, shaded, and nutrient-poor environments^1^. Most clonal plants possess the capacity for both sexual reproduction through seeds and clonal propagation (asexual reproduction) through bud banks^1^,^2^. Both modes of reproduction contribute to population persistence of clonal plants^3^,^4^,^5^. Sexual reproduction enables long-distance seed dispersal, reduces local intraspecific competition, and ensures genetic diversity^5^. In contrast, clonal propagation mainly contributes to local population growth and high resilience following herbivory, drought, and other stresses^6^.

Clonal plants allocate resources to sexual reproduction and vegetative propagation from the same resource pool during a reproductive episode^7^. Resource allocation to the two modes of reproduction is influenced by a variety of biological factors such as plant size and population age^8^,^9^, and abiotic variables such as nutrient level and successional status^6^,^7^,^10^. Loehle predicted that clonal plants should increase sexual reproduction in favorable site conditions^11^, whereas other studies have suggested that clonal plants increase asexual reproduction in stable or productive surroundings^12^,^13^.

Theoretically, increased allocation toward one function should result in a reduction in investment in the other function. Allocation trade-offs between vegetative and sexual reproduction have been documented in various clonal species^7^,^10^,^14^, whereas little evidence has been found in other species^15^,^16^,^17^,^18^. Nevertheless, most of these studies were theoretic models or manipulated experiments in which the effort a plant makes to a certain function was steered to reveal the relative importance of the reproductive functions^5^. Only few studies have investigated reproductive allocation in clonal plants in field conditions with variable environmental factors.

In this study, we investigated the trade-off between allocation to sexual reproduction and clonal propagation in the wetland sedge *Carex brevicuspis* C. B. Clarke across a small-scale elevational gradient (21–27 m a.s.l.) at the Dongting Lake wetlands, China. *C. brevicuspis* is a typical rhizomatous clonal plant that is widely distributed in the study area^19^,^20^. It reproduces sexually through seeds and asexually via rhizome buds^21^,^22^. In freshwater wetlands or floodplains, the elevation, which closely reflects the hydrological and edaphic conditions at which plants occur, is the most important factor affecting plant growth, reproduction, and distribution^20^,^23^,^24^,^25^,^26^. For wetland sedges, low-elevation sites may represent harsh conditions for plant growth and reproduction due to the longer duration of flooding submergence and the irregular flooding during the growing season^20^,^27^. In contrast, high-elevation sites may provide favorable conditions for plant growth and reproduction due to the longer growing season and aerated soil^20^.

We addressed the following two hypotheses: (1) more reproductive ramets would be produced at low-elevation sites where habitat conditions are harsh, whereas more vegetative buds would be produced by plants at high-elevation sites where conditions are relatively favorable; and (2) there would be a trade-off between sexual reproduction and vegetative propagation, i.e., an increase in allocation to sexual reproduction would decrease the allocation to vegetative propagation, and vice versa. To test these hypotheses, we investigated the demography of rhizome buds, and vegetative and reproductive ramets of *C. brevicuspis* by sampling belowground buds and aboveground shoot populations, and recorded environmental factors over one complete growing season at three elevations (low, 21–23 m; intermediate, 24–25 m; high 25–27 m) in the Dongting Lake wetlands.

## Results

### Biomass and biomass allocation

**Figure 1:**
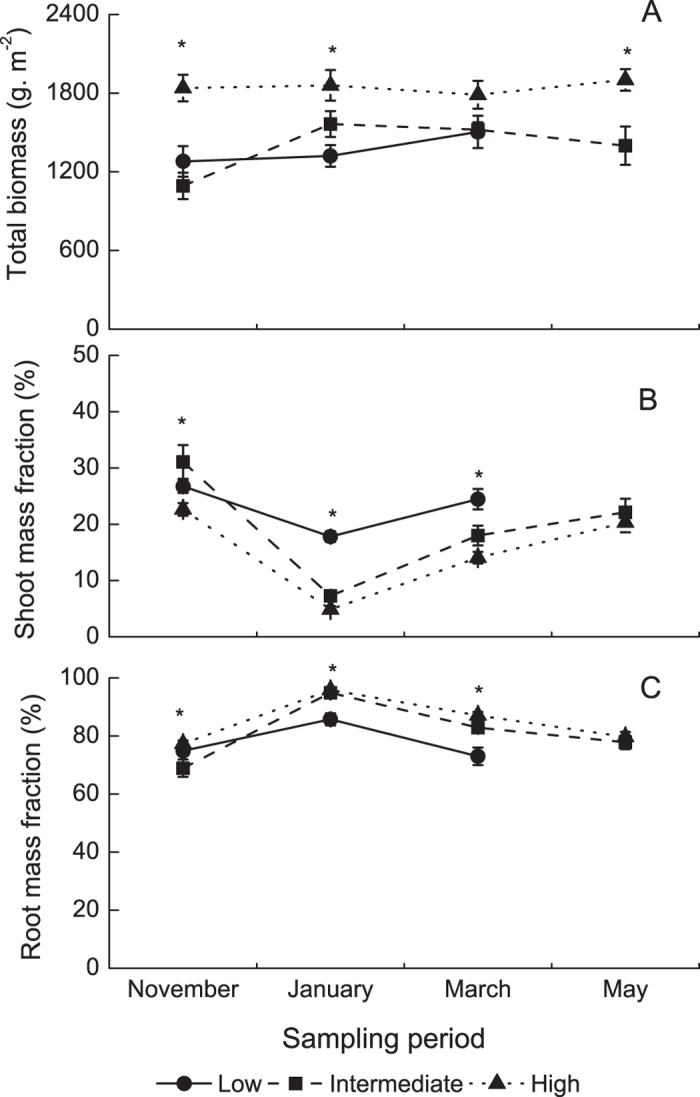
Plant biomass (**A**), shoot mass fraction (**B**), and root mass fraction (**C**) at three elevations at the four sampling time points. The data are expressed as the mean ± SE. *P < 0.05.

The total plant biomass was significantly affected by elevation (Table 1), with higher biomass at high elevations (1787.23–1900.91 g·m^−2^) than at low and intermediate elevations (1092.19–1563.90 g·m^−2^) (Fig. 1A). The shoot and root mass fractions were significantly affected by elevation and sampling period, with significant interactions (Table 2). The shoot mass ratio was higher at low elevations in January and March (17.81 ± 0.96% in Jan and 24.47 ± 1.81% in Mar) than at intermediate (7.28 ± 0.61% in Jan and 18.00 ± 1.76% in Mar) and high elevations (4.87 ± 0.58% in Jan and 18.00 ± 1.03% in Mar) (Fig. 1B). The root mass ratio was lower at low elevations in January and March (85.75 ± 2.08% in Jan and 73.01 ± 3.03% in Mar), and higher at intermediate (94.91 ± 1.22% in Jan and 82.88 ± 1.88%) and high elevations (95.86 ± 0.67% in Jan and 87.12 ± 1.18% in Mar) (Fig. 1C).

### Ramet and bud densities

**Figure 2:**
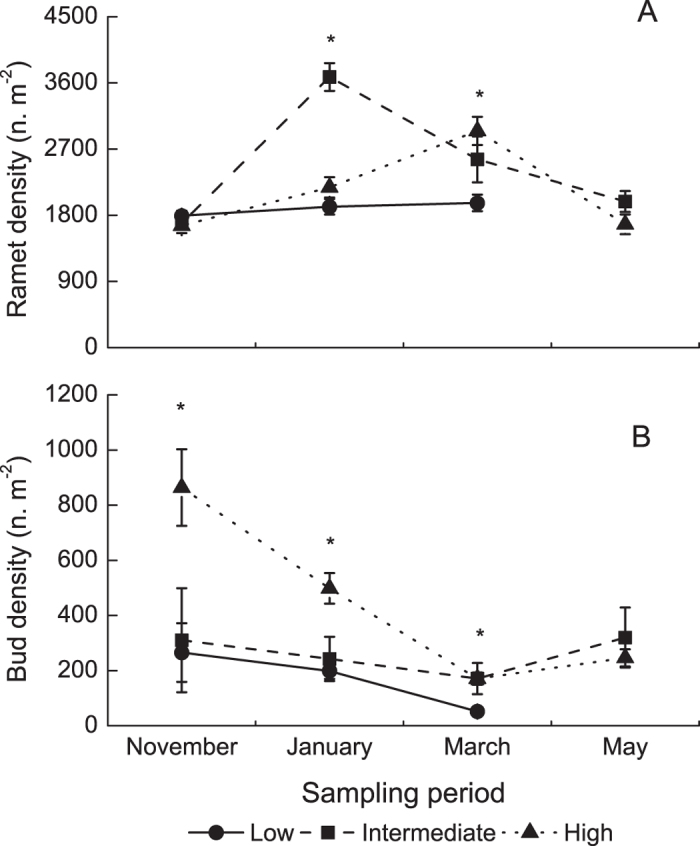
Ramet density (**A**) and bud density (**B**) at three elevations at the four sampling time points. The data are expressed as the mean ± SE. *P < 0.05.

The ramet and bud densities were significantly affected by elevation and sampling period, with significant interactions (Table 1). The ramet density was higher at intermediate elevation (3680 ± 189 ramets m^−2^) than at high and low elevations (2179 ± 139 and 1916 ± 106 ramets m^−2^, respectively) in January, while the ramet density was higher at high and intermediate elevations (2947 ± 192 and 2560 ± 312 ramets m^−2^, respectively) than at low elevations (1968 ± 113 ramets m^−2^) in March (Fig. 2A). The bud density was the highest at high elevations in November and January (864 ± 139 buds m^−2^ in Nov and 498 ± 55 buds m^−2^ in Jan), intermediate at intermediate elevations (301 ± 189 buds m^−2^ in Nov and 243 ± 80 buds m^−2^ in Jan), and the lowest at low elevations (266 ± 106 buds m^−2^ in Nov and 199 ± 29 buds m^−2^ in Jan) (Fig. 2B).

### Densities and biomass of reproductive ramets, vegetative ramets, and rhizome buds in March

**Figure 3:**
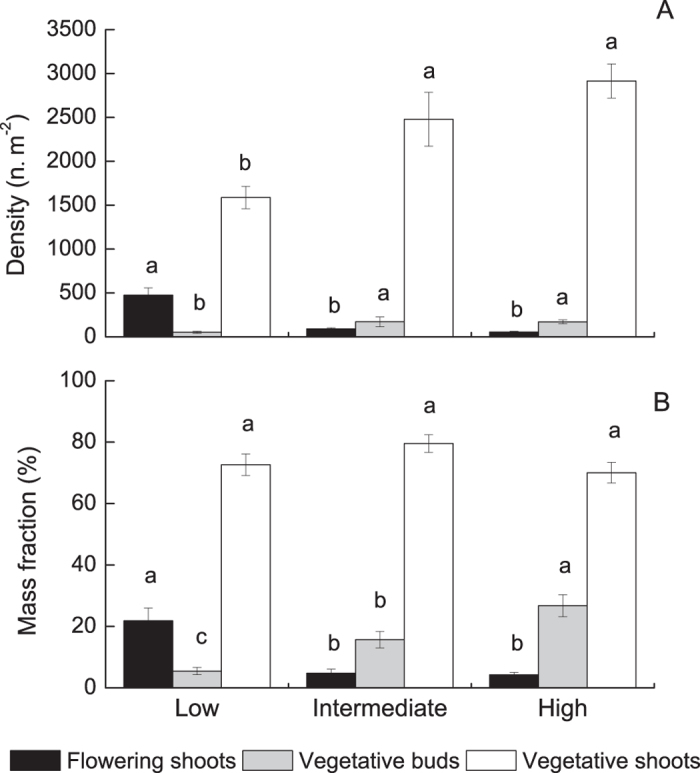
The density (**A**) and mass fraction (**B**) of reproductive ramets, rhizome buds, and vegetative ramets at three elevations in March at the Dongting Lake wetlands. Different letters indicate differences at the 0.05 significance level (Tukey’s test).

The densities of reproductive ramets, vegetative ramets, and rhizome buds were significantly affected by elevation in March (Fig. 3A). The density of reproductive ramets was higher at low elevations (475 ± 83 ramets m^−2^) than at intermediate and high elevations (91 ± 13 and 55 ± 9 ramets m^−2^, respectively) (Fig. 3A). In contrast, the density of vegetative ramets was lower at low elevations (1588 ± 128 ramets m^−2^) than at intermediate and high elevations (2478 ± 307 and 2914 ± 194 ramets m^−2^, respectively). The density of rhizome buds was lower at low elevations (52 ± 11 buds m^−2^) than at intermediate and high elevations (170 ± 57 and 171 ± 22 buds m^−2^, respectively).

The proportion of biomass allocated to reproductive ramets was higher at low elevations (21.85 ± 4.14%) than at intermediate and high elevations (4.77 ± 1.35% and 4.28 ± 0.73%) (Fig. 3B). In contrast, the proportion of biomass allocated to rhizome buds was the lowest at low elevations (5.48 ± 1.20%), intermediate at intermediate elevations (15.69 ± 2.68%), and the highest at high elevations (26.75 ± 3.60%).

### Relations between reproductive allocation and environmental factors

Generally, the SM and soil nutrient contents and the pH were higher at low than at high elevations in three sampling points (Table 2). In March, the SM, SOM, and TP contents and pH were higher at low than at high elevations (Table 2). The SBD was lower at low elevations than at intermediate and high elevations, while the TK was higher at intermediate than at high elevations.

**Figure 4:**
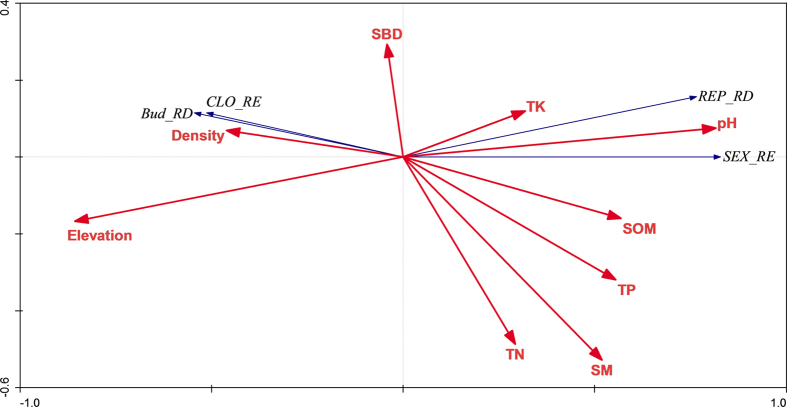
Redundancy analysis (RDA) biplots showing the relationships between reproductive allocation and environmental variables in Dongting Lake wetlands. SM, soil moisture content; SBD, soil bulk density; SOM, soil organic matter; TN, total nitrogen; TP, total phosphorus; TK, total potassium; SEX_RE, mass fraction to reproductive ramets; CLO_RE, mass fraction to rhizome buds; REP_RD, the relative density of reproductive ramets; BUD_RD, the relative density of rhizome buds.

The first axes of the RDA ordination explained 68.6% of the total variance of the species-environment relationship (Table 3). The first axis was positively correlated with SM, SOM, TP, and pH and negatively correlated with elevation and ramet density (Fig. 4).

## Discussion

*C. brevicuspis* produces vegetative ramets and rhizome buds during the entire growing season, whereas it produces reproductive ramets only in spring. In March, *C. brevicuspis* produced more reproductive ramets at low-elevation sites than at intermediate and high elevations. This result was consistent with our first hypothesis, which predicted that plants allocate more resources toward sexual reproduction at low-elevation sites.

*C. brevicuspis* significantly invests in sexual reproduction through flowering and seed production, particularly at low-elevation sites. However, population recruitment of *C. brevicuspis* occurs from belowground buds rather than from seeds^21^,^22^,^25^. Clonal species, for which local recruitment mainly depends on vegetative propagation, allocate more nutrients to sexual reproduction when the ramet density becomes high, because the additional clonally produced individuals have a low probability of survival in the local area^3^,^28^. Sun *et al*. found that sexual reproduction in *Scirpus mariqueter* increased with ramet density from low to high elevation in estuary marshes^7^. In contrast, the present study showed that sexual reproduction in *C. brevicuspis* decreased with increasing ramet density. Our results are consistent with Gardner and Mangel’s model that clonal species should have adapted to competition and decrease sexual reproduction at higher densities^13^, as reported in previous studies^29^,^30^.

Specific habitat conditions may account for differences in reproductive allocation patterns among studies. In the Dongting Lake wetlands, the submergence duration decreases with increasing elevation^25^,^27^. In addition, *C. brevicuspis* populations at low-elevation sites are subject to irregular flooding caused by regional precipitation, which probably causes death of the shoot population^25^. These characteristics indicate that low-elevation habitats are prone to flood disturbances whereas high-elevation sites are relatively stable. In disturbed environments, clonal plants may allocate more resources to sexual reproduction as a survival mechanism^4^,^31^. Seeds produced at low-elevation sites may also have a greater chance of dispersal by floodwater and of establishment in bare beaches^22^.

The ordination produced by RDA indicated that allocation to sexual reproduction positively correlated with SM and nutrient contents and negatively correlated with ramet density and elevation. These results suggest that the allocation to sexual reproduction is favored in low-elevation habitats (frequently disturbed by flooding) and nutrient-rich environments, whereas the allocation to asexual reproduction is favored in high-elevation habitats (less frequently disturbed by flooding) and competitive environments (high ramet density). Future studies should be conducted to clarify the relative importance of these two categories of selective forces on the evolution of reproductive strategies of clonal plants.

*C. brevicuspis* allocated more biomass to sexual than to vegetative propagation at low-elevation sites, while it allocated more biomass to vegetative than to sexual reproduction at high-elevation sites. These results supported our second hypothesis, which predicted a trade-off between investment in sexual reproduction and vegetative propagation in *C. brevicuspis*.

Allocation trade-offs between vegetative and sexual reproduction have been well documented in various clonal plant species^7^,^10^,^14^. However, not all plants exhibit such trade-offs in their life-history strategies^5^. For example, *Andropogon gerardii* can increase flowering and seed production and simultaneously produce more buds^6^. Trade-offs between two reproductive modes seem to occur when resources are limited^5^,^10^. Shoot emergence and vegetative propagation in *Carex* species are often limited by the carbohydrate reserves stored during the reproductive episode^21^,^32^. At low-elevation sites, *C. brevicuspis* may allocate a large proportion of energy and nutrients to inflorescence development rather than to rhizome formation^32^. In contrast, *C. brevicuspis* may allocate more energy and nutrients to the production of rhizome buds at high-elevation sites. Internal competition between sexual and asexual reproductive traits may result in less sexual reproduction when resources are limited^9^.

## Conclusion

Our results demonstrated that sexual reproduction of *C. brevicuspis* decreased, whereas vegetative propagation increased with elevation. A trade-off between sexual reproduction and vegetative propagation exists during the reproductive episode of *C. brevicuspis*. Our findings suggested that the allocation to sexual reproduction is favored in disturbed habitats with fertile soils, whereas the allocation to vegetative propagation is favored in stable and competitive habitats. Trade-off between allocation to sexual and vegetative reproduction along an elevational gradient might be a reproductive strategy of *C. brevicuspis* to adapt to the dynamic wetland habitats.

## Methods

### Study species

*Carex brevicuspis* (Cyperaceae) is a perennial rhizomatous sedge distributed in eastern mainland China and Taiwan^33^. The pseudoculm of the plant, made up of a series of overlapping leaf sheaths, is usually 20–55 cm in height. In the Dongting Lake wetlands, this species forms mono-dominant communities or is co-dominant with other *Carex* species. During the flood season, *Carex* vegetation is completely submerged and aboveground shoots senesce. *C. brevicuspis* shoots emerge immediately after flooding (November) and grow into a standing crop before January^21^. In January, plants remain relatively dormant, but shoots partially wither because of low temperature. New ramets sprout in March, after which plants grow rapidly. Plants flower and fruit from March to May, but seedlings are scarce in the field^21^,^22^.

### Study sites

Dongting Lake (28°30′–30°20′N, 111°40′–113°10′E), the second largest freshwater lake in China, is located in the northern Hunan Province. It lies in a basin to the south of the Yangtze River, and is connected to the Yangtze by distributary channels. The surrounding wetlands are characterized by large seasonal fluctuations in the water level (up to 15 m); they are completely flooded from June to October and exposed from November to May. The mean annual temperature is 16.8 °C, with hot summers (June–August, 27.3 °C) and cold winters (December–February, 5.8 °C)^34^. Annual precipitation is 1382 mm, with more than 60% falling between April and August.

We used three sections of the lake shoreline where *C. brevicuspis* is extensively distributed as study sites, including Matang (29°14′17.6″N, 113°03′46.6″E), Junshan (29°22′29.5″N, 113°00′31.0″E), and Dingzidi (29°25′47.9″N, 112°56′46.7″E). *C. brevicuspis* is usually distributed adjacent to the water body and extends to the embankment, along a mild slope of 5–10° 20, which provides an ideal elevation gradient for this investigation.

### Elevation gradient and sample transects

We separated the elevation gradient into three categories: high (25–27 m), intermediate (24–25 m), and low (21–23 m) elevation. In total, nine transects (150 × 20 m; one transect per elevation per site) running parallel to the water body were established. At each site, the transects were 500 m apart.

### Sampling methods

Ramets of *C. brevicuspis* were sampled immediately after flooding (November), in winter (January), in spring (March), and before flooding (May) using a permanent plot sampling method^20^. In November 2013, five permanent plots (2 × 2 m) were established 100 m apart on each transect at each site, in areas where *C. brevicuspis* formed mono-dominant communities. The corners of each plot were marked with durable plastic tubes hammered into the soil. On each sampling occasion, one square (25 × 25 cm) was randomly excavated in each permanent plot, giving a total of 45 squares per sampling occasion. In each square, all living (>50% green, potentially photosynthetically active) aboveground shoots were clipped and placed in plastic bags. Undisturbed soil within the squares was excavated to a depth of 15 cm using a shovel and stored in plastic bags^21^,^25^. In addition, from an area adjacent to each square, two soil samples were collected to a 10-cm depth by using cutting rings (100 cm^3^): one was used for measuring the soil moisture (SM) content and bulk density (SBD), and the other was used for determining the soil organic matter (SOM), total nitrogen (TN), total phosphorous (TP), total potassium (TK) contents, and pH. All samples were immediately transported to the laboratory for processing. The low-elevation sites were unavailable for plant and soil sampling in May because they were submerged by early floods.

### Sample processing

Aboveground shoots were separated into vegetative and reproductive shoots and counted separately. Belowground tissue samples were carefully washed to remove the soil while protecting the integrity of the rhizomes. For each sampled square, the numbers of reproductive ramets, vegetative ramets, and rhizome buds were counted. Vegetative shoots, reproductive shoots, roots, and rhizomes were dried separately in an oven at 80 °C for 48 h before obtaining the dry weight. The plant total biomass was defined as the total dry weight of vegetative shoots, reproductive shoots, roots, and rhizomes per m^2^. The density and biomass of reproductive ramets served as indices of sexual reproduction effort, while the density and biomass of rhizomes buds were considered indices of clonal reproduction effort^7^.

All procedures for determining soil variables were in accordance with the Chinese national standards^35^. SM was determined after drying the samples in an oven at 105 °C for 48 h. SM was calculated as ([W − D]/W) × 100%, where W is the soil fresh weight and D is the soil dry weight. The remaining fresh soil samples were air-dried in the shade and sieved through a 20- or 60-mesh screen for analysis of the other soil variables. The pH was measured using a pH meter (Delta 320; Mettler-Toledo, Greifensee, Switzerland) at a soil:distilled water ratio of 1:2.5. We determined the SOM by wet digestion using the Walkley-Black method. TN was digested by the micro-Kjeldahl method and measured using a flow injection analyzer (FIAstar 5000, FOSS, Hillerød, Denmark). TP was digested with sodium hydroxide and measured colorimetrically using the ascorbic acid reduction method. TK was digested with sodium hydroxide and measured using an atomic absorption spectrometer (932AA; GBC, Melbourne, Australia). All soil analyses were conducted at the Key Laboratory of Agro-ecological Processes in the Subtropical Region of the Chinese Academy of Sciences.

### Data analysis

We tested the significance of differences in the density and biomass of ramets and rhizome buds, and shoot and root mass fractions at different elevations and during different sampling periods using linear mixed models, with elevation and sampling period included as main factors and sampling site as a random factor^36^. *C. brevicuspis* produced reproductive ramets only in March; therefore, we tested the significance of differences in the densities and fractions of total biomass of reproductive ramets, vegetative ramets, and rhizome buds at different elevations in the data of March using linear mixed models, with elevation included as a main factor and sampling site as random factor. Multiple comparisons of means were performed using Tukey’s honestly significant difference (HSD) test at the 0.05 significance level. The data were square root- or log_10_-transformed, if necessary, to reduce the heterogeneity of variances, and the homogeneity was tested using Levene’s test. The relationships between reproductive allocation and environmental variables in the data of March were investigated by multivariate analysis. The relationships were resolved to the best level by redundancy analysis (RDA) based on several iterations. The input vegetation data matrix consisted of the relative density and biomass of reproductive ramets and rhizome buds of *C. brevicuspis* to total density and total biomass respectively, and the environmental data matrix consisted of elevation, soil variables, and ramet density. Total density was defined as the sum density of rhizome buds, and vegetative and reproductive ramets. RDA was conducted using CANOCO version 4.5 (Plant Research International, Wageningen, the Netherlands). Species-environment ordination diagrams were prepared with CanoDraw LITE to illustrate the results^37^. The data are expressed as the mean ± the standard error (SE), and a *P*-value < 0.05 was considered significant.

## Additional Information

**How to cite this article**: Chen, X.- *et al*. Trade-off between allocation to reproductive ramets and rhizome buds in *Carex brevicuspis* populations along a small-scale elevational gradient. *Sci. Rep*. **5**, 12688; doi: 10.1038/srep12688 (2015).

## Figures and Tables

**Table 1:** Linear mixed model analysis (*F*-values) of ramet and bud densities, total biomass, and biomass allocation.

Variable	Elevation (E)	Sampling period (S)	E × S
Ramet density	11.06^***^	25.35^***^	12.39^***^
Bud density	20.07^***^	24.61^***^	10.49^***^
Total biomass	17.66^***^	2.11^ns^	1.71^ns^
Shoot mass fraction	15.30^***^	53.92^***^	4.57^**^
Root mass fraction	10.41^***^	52.94^***^	5.00^***^
d.f.	2	3	6

Elevation and sampling period were included as fixed factors, and site was included as a random factor.

^***^P < 0.001, ^**^P < 0.01^, *^P < 0.05; ^ns^*P* > 0.05.

**Table 2:** Soil characteristics at each elevation in the study area in three sampling periods.

Sampling period	Elevation category	SM (%)	SBD (g·cm^−3^)	SOM (%)	TN (%)	TP (%)	TK (%)	pH
2013.11	Low	0.66 ± 0.04a	0.70 ± 0.03b	5.05 ± 0.27a	0.24 ± 0.01a	0.09 ± 0.00a	0.18 ± 0.00a	7.50 ± 0.12a
	Intermediate	0.61 ± 0.01ab	0.85 ± 0.02a	4.41 ± 0.18a	0.21 ± 0.01b	0.09 ± 0.01a	0.15 ± 0.01b	5.95 ± 0.15b
	High	0.56 ± 0.02b	0.75 ± 0.05ab	4.82 ± 0.30a	0.22 ± 0.01ab	0.07 ± 0.00b	0.15 ± 0.00b	5.99 ± 0.15b
2014.1	Low	0.68 ± 0.03a	0.74 ± 0.04c	4.20 ± 0.27a	0.22 ± 0.02a	0.09 ± 0.00a	0.16 ± 0.00a	7.10 ± 0.09a
	Intermediate	0.46 ± 0.03b	1.07 ± 0.03a	3.25 ± 0.06b	0.22 ± 0.01a	0.08 ± 0.00b	0.15 ± 0.01b	6.39 ± 0.21b
	High	0.54 ± 0.02b	0.90 ± 0.03b	3.77 ± 0.16ab	0.20 ± 0.01a	0.06 ± 0.00b	0.14 ± 0.00b	5.78 ± 0.09c
2014.3	Low	0.86 ± 0.06a	0.65 ± 0.02b	4.24 ± 0.11a	0.20 ± 0.01a	0.08 ± 0.00a	0.15 ± 0.00a	7.19 ± 0.07a
	Intermediate	0.66 ± 0.05b	0.91 ± 0.04a	2.93 ± 0.17b	0.19 ± 0.01a	0.07 ± 0.00a	0.16 ± 0.00a	6.28 ± 0.10b
	High	0.64 ± 0.03b	0.90 ± 0.03a	2.96 ± 0.08b	0.20 ± 0.01a	0.06 ± 0.00b	0.15 ± 0.00a	6.00 ± 0.12b

SM: soil moisture content; SBD: soil bulk density; SOM: soil organic matter; TN: total nitrogen; TP: total phosphorus; TK: total potassium. The data are expressed as the mean ± SE. Different letters indicate significant differences between elevations (*P* < 0.05, Tukey’s HSD test).

**Table 3:** Summary of the redundancy analysis (RDA) ordinations.

Axes	Axis 1	Axis 2
Statistics		
Eigenvalues	0.686	0
Species-environment correlations	0.828**	0.371*
Cumulative variance (%)	68.6	68.6
Elevation	−0.710**	−0.062
SM	0.439**	−0.196
SBD	−0.035	0.109
SOM	0.471**	−0.059
TN	0.242	−0.181
TP	0.459**	−0.119
TK	0.264	0.045
pH	0.676**	0.028
Ramet density	−0.382**	0.026

SM: soil moisture content; SBD: soil bulk density; SOM: soil organic matter; TN: total nitrogen; TP: total phosphorus; TK; total potassium. Percentage variance of species-environment relationships explained by the first two ordination axes. Correlations indicate intra-set correlations of environmental factors with the first two ordination axes. *P  < 0.05; **P < 0.01.
